# Betel Quid Use and Tuberculosis Transmission: A Neglected Focus Area for Tuberculosis Control in Low- and Middle-Income Countries

**DOI:** 10.1093/ofid/ofae577

**Published:** 2024-10-05

**Authors:** Priyanka Manghani, Narayana Prasad, Nishtha Khatri, Robert Paulino-Ramirez, Shishir Gokhale, K M Monirul Islam, Piyusha Majumdar, Tran Hoang, Hanifa Denny

**Affiliations:** California Rural Indian Health Board, Public Health Literacy, 1020 Sundown Way, Roseville, CA 95661, USA; Cardiovascular Imaging and Core Lab, Brigham and Women´s Hospital, Public Health Literacy, 75 Francis St, Boston, MA 02115, USA; Division of Holistic Health, Mahati Wellness, Bandara West, Mumbai 400 050, India; Instituto de Medicina Tropical & Salud Global, Universidad Iberoamericana (UNIBE), UNIBE Research Hub, Santo Domingo 22333, Dominican Republic; Department of Microbiology, Manipal College of Medical Sciences, Pokhara, Nepal; Public Health Literacy, 7282 SW 23 St, Miami, FL 33172, USA; Indian Institute of Health Management and Research, Prabhu Dayal Marg, Jaipur-302029, India; FHI 360, 2101 L St NW, Suite 700, Washington, DC 20037, USA; Occupational Health and Safety, Faculty of Social and Political Sciences, Universitas Diponegoro, Jl. Dr. Antonius Suroyo, Semarang City, Central Java 50275, Indonesia

**Keywords:** betal, chewing, determinants, epidemiology, tuberculosis

## Abstract

Habitual betel quid consumption and spitting contribute to tuberculosis (TB) transmission due to direct exposure to pathogens, immunosuppression, and social contact. Despite betel quid being classified as a group 1 human carcinogen and a high prevalence of betel quid consumption in patients with TB, there exists a knowledge gap in the relationship between quid use and TB, which presents as a neglected opportunity to address the global burden of TB in low- and middle-income countries. Understanding such a knowledge gap is crucial when taking measures at various levels, including research prioritization, behavior change communication, and legislation to address the availability and access of quid products, coupled with community-based interventional strategies. This article thus presents empirical evidence on quid use and its effects on TB spread and identifies feasible, applicable changes required at various levels to curtail the spread of TB among betel quid users.

Betel quid, popularly known as *paan*, is a popular chewing product composed of the nut of the areca palm, the leaf of the betel pepper (*Piper betel*), and a tint of lime (calcium hydroxide) [1]. It is the fourth-most addictive substance after alcohol, nicotine, and caffeine, and it is a group 1 oral carcinogen, consumed by >600 million people globally [2, 3].

There is a large volume of scientific evidence linking betel and areca nut consumption with an increased risk of oral cancers. A meta-analysis of 15 case-control studies showed that betel quid without tobacco has an independent positive association with oral cancer due to the carcinogenic potential of the areca nut, with an odds ratio (OR) of 2.82 (95% CI, 2.35–3.40; *P* < .001) [4]. The burden due to betel-induced oral cancer is quite large, and a study conducted in Taiwan assessing the risk of oral cancer in high-risk individuals found that those consuming betel nuts were 2.12 times more likely to develop oral cancer, with smoking as a baseline for comparison (hazard ratio, 2.12); thus, chewing betel nut had a significantly higher oral cancer incidence risk when compared with only a habit of smoking [5]. Nearly 50% of oral cancers occurring in regions such as the Indian subcontinent and Taiwan are attributed to betel quid chewing (population attributable fraction, 53.7% for residents in Taiwan and 49.5% for the Indian population), which highlights the need to address this modifiable risk factor [6].

Arecoline, one of the alkaloids found in the areca nut component of betel quid, is parasympathetic in action and leads to salivary glandular secretion when one is chewing the quid producing large volume of saliva as well as quid [7, 8]. Spitting the quid can lead to the potential spread of communicable diseases due to the possibility of person-to-person transmission of tuberculosis (TB) owing to the communal environment of quid chewers, spitting of saliva, and exposure to pathogens through salivary droplets [8]. However, this potential association is often ignored in the TB spread pathway as compared with traditional cigarette smoking, which is known to increase the risk of TB while contributing to poor treatment outcomes [9]. Thereby, the aim of this review article is to address the ignored association between betel quid consumption and the transmission of TB—especially since there has been a rising prevalence of betel quid consumption in Asia, with high betel quid use disorder being reported across endemic Southeast Asian countries, such as India, Pakistan, and Sri Lanka (8.4%–40%); followed by Nepal, Malaysia, and Indonesia (10.3%–47.8%); China and Taiwan (2.3%–29%); and Palau and Solomon Islands (72%–83%) [10, 11]. This recent increase in betel quid consumption has contributed to the easy availability of betel quid and its low cost as compared with cigarettes, along with the social acceptance of quid chewing [10, 12]. Considering that this geographic region accounts for 45% of the global TB burden and 81% of the global TB deaths, the possibility of a stronger association between betel quid and TB should be explored [13, 14].

A study in Cambodia found a strong association between betel quid use and TB (OR, 4.43; 95% CI, 1.66–11.86) [8]. A study in Indonesia reported that among patients with active TB and betel chewing, there was higher TB transmission owing to betel-chewing culture [15]. Furthermore, a study conducted in Northern India revealed a high prevalence of current tobacco consumption in incident TB cases, with 10.7% of 211 cases being active consumers of smokeless tobacco [16]. In lieu of these findings, our article explores the current empirical evidence available on betel quid use and its effects on TB spread and identifies feasible, applicable intervention strategies required at various levels to curtail the spread of TB among betel quid users.

## PATHOLOGIES LINKED TO BETEL CONSUMPTION

Betel nut consumption has been associated with an increased risk of a variety of malignant conditions, among other pathologies.

Betel nut has been classified as a group 1 carcinogen by the International Agency for Research on Cancer [10]. Given this, most evidence on the health effects of betel have been focused on oral and other cancers and its impact on cardiovascular health and cerebrovascular diseases [17, 18]. The alkaloids of the areca nut are its prime carcinogenic ingredient, and areca nut in any form contributes to oral cancer, with cancers of the esophagus, liver, pancreas, larynx, and lungs becoming fairly common in areca nut users as well [10, 19, 20]. Habitual betel quid chewing contributes to adverse cardiovascular outcomes. It is also a risk factor for arrhythmias and premature ventricular contractions [21, 22].

A systematic review revealed that habitual betel quid chewing was associated with hypertension, atherosclerosis, inflammation, and ischemic heart disease in addition to being an independent risk factor for cardiovascular disease in women [23]. Areca nut also has an association with systemic health conditions, including diabetes, metabolic syndrome, and hepatocellular carcinoma [24–27].

There has been evidence showing the impact of areca nut consumption on respiratory pathologies such as asthma. A case-control study in Taiwan revealed a significant association between betel consumption and asthma (adjusted OR, 2.05; 95% CI, 1.12–3.76) [28].

It is important to recognize that excessive consumption of betel nut can lead to addiction. Furthermore, the habit of chewing betel nut can spread among individuals through social interactions, contributing to a wider prevalence of betel nut addiction [29].

## BETEL USE AND TB: GAPS IN EPIDEMIOLOGIC EVIDENCE

Despite the evidence showing an association between betel consumption and cardiovascular diseases and respiratory and oncology pathologies, there is limited evidence on the possible association of betel quid consumption and TB. This evidence is also limited as compared with the empirical evidence available on smoking and tobacco, where smoking is a known risk factor for TB and a contributor to poor treatment outcomes [9]. Studies found a strong association between betel quid and TB (OR, 4.43; 95% CI, 1.66–11.86) [8]. However, no causality could be determined and this association was based on a small group of prevalent TB cases (8 of 63 betel quid users), which do not make the results generalizable [8]. Yet, the study highlighted the possibility of an increased risk of TB infection in quid users with several factors contributing to the pathophysiology of TB in these cases, such as person-to-person transmission, spread through spitting of quid saliva, and immunosuppression [8]. Similarly, a study conducted in Indonesia revealed that of 51.8% (n = 57) of patients who had active TB, the TB transmission was higher when there was active TB and betel chewing present [15].

Areca nut chewers are predisposed to asthma owing to increased bronchoconstriction and a decreased forced expiratory volume in the first second [17]. A case-control study in India found that a history of asthma was associated with an increased risk of developing pulmonary TB, with 74.5% of positive TB cases having a history of asthma [30]. However, there is poor evidence linking the increased risk of TB in patients with asthma. A case-control study in the United Kingdom reported a slight amount of risk for TB in patients with asthma, while 3 studies conducted in Western Africa concluded that history or treatment of asthma had no effect on the risk for TB [31, 32]. In addition to asthma, areca nut was positively correlated with chronic obstructive pulmonary disease and lung function impairment [33]. This could be linked to eotaxin 1 activation and chronic inflammation, and chronic obstructive pulmonary disease is a known risk factor for pulmonary TB [34, 35].

Areca nut leads to suppression of T-cell activity and decreased release of cytokines, thus affecting the immune system [17]. This could contribute to the risk for TB, since TB is common in persons who are immunocompromised and the adaptive immune response mediated by T cells is critical for control of *Mycobacterium tuberculosis* infection in humans [36, 37]. Furthermore, betel increases the risk of type 2 diabetes mellitus, which is a known risk factor for the development of TB [38, 39].

**Figure 1.**
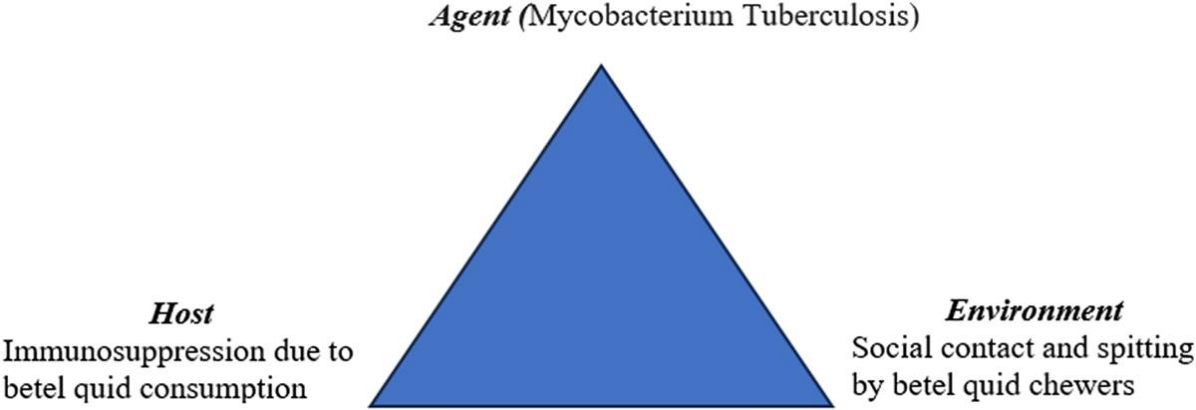
Epidemiologic triad for the potential association between tuberculosis and betel quid use proposed by the authors.

While these evidences outline a possible linkage of betel quid consumption, immunosuppression, and TB risk, there remain gaps in evidence regarding the direct impact of betel quid consumption on individual risk and susceptibility for pulmonary TB, with no robust scientifically researched data that offer insight into its effect. Figure 1 displays an epidemiologic triad to explain this potential association, while Table 1 presents the empirical findings of epidemiologic studies describing the association between betel quid use and TB.

**Table 1.** Current Epidemiologic Evidence on the Association Between Betel Quid Consumption and Tuberculosis

Study	Country	Year	Design	Results	Limitations
“Betel Quid Use in Relation to Infectious Disease Outcomes in Cambodia” [8]	Cambodia	2012	Cross-sectional study	An association between the intensity of betel quid use and TB (OR, 1.50; 95% CI, .96–2.36) with a strong association between betel quid use and TB in men (OR, 4.43; 95% CI, 1.66–11.86).	Prevalent cases and results cannot be generalized and no causality.
“Factors Influencing Transmission of Tuberculosis in Ngeu Nata Culture among Ngada Community in Kupang, East Nusa Tenggara, Indonesia” [15]	Indonesia	2021	Cross-sectional study	High TB transmission behavior in betel-eating respondents (51.8%)	Results cannot be generalized and no causality.

Abbreviations: OR, odds ratio; TB, tuberculosis.

## SOCIOCULTURAL FACTORS INFLUENCING BETEL USE

Betel quid is a form of smokeless tobacco, and its use has been an important part in various South Asian cultures, strongly influenced by social acceptability, traditional medicine, as well as religious beliefs [40, 41]. Betel has been used in South Asian culture for functions such as weddings and reconciliation ceremonies, as well as in traditional medicine as an antiseptic and breath freshener [41, 42]. Areca nut is one of the major constituents of betel quid and is considered a vital ingredient in religious ceremonies; its consumption in betel quid is also related to its euphoric stimulation property owing to the high levels of psychoactive alkaloids present in it [19, 43].

Chewing of areca nut has been shown to increase work capacity, causing hot sensations and fueling alertness [43]. Some of the common reasons why users claimed that they chewed areca nut was the refreshing feeling that it gave with its good taste or as a snack to relieve stress and/or strengthen gums [44].

The high prevalence of consuming betel in Southeast Asia is influenced by factors such as age, gender, education, and socioeconomic disparities in purchasing power. Since betel is a form of smokeless tobacco, many advertising campaigns do not outline the health risks of betel as compared with cigarette smoking [45]. A qualitative study examining perceptions of adolescents toward areca nut revealed that adolescents view tobacco as a higher-risk substance due to the greater focus on tobacco harms through various media portals [46]. Additionally, since areca nut is marketed as a sweeter substance, many adolescents do not perceive it as a harmful substance to health, and it is often consumed for a boost of energy [46]. In many regions, cigarette smoking is more prevalent in men and is not considered socially acceptable for women; hence, areca nut consumption is more prevalent among women and adolescent girls owing to greater social acceptability [46, 47]. Last, betel has easy availability coupled with an attractive price point and misleading advertisements as compared with cigarettes, which further influence its consumption in society [12].

## POSSIBLE SUGGESTIONS TO CONTAIN TB SPREAD AMONG BETEL USERS

### Focus on Prevention, Health Education, and Integration Into National TB Control Programs

There is poor awareness and literacy regarding the health impacts of betel quid as compared with cigarettes. This can be attributed to factors such as misrepresentation and the false perception that betel has potentially less negative health impacts as compared with cigarettes [47]. Addressing this false perception is crucial, which highlights the need for health literacy programs. Health promotion programs should focus on increasing public literacy of the negative health impacts of smokeless tobacco products such as betel. Much like the measures incorporated to address the negative health consequences in cigarette advertisements, similar regulations could be improvised to spread information regarding the health impacts of betel. This initiative requires multistakeholder engagement, including policy makers and government agencies. Addressing the negative health impacts of betel quid would be particularly challenging in low socioeconomic groups and rural areas, where a far more intensive effort would be required, with health promotion plans having to be culturally sensitive. Such initiatives should be designed with respect to a sociocultural context in a diverse society such as Southeast Asian countries. Integrating these initiatives into national TB control programs is an option, but the successful implementation remains a daunting task. Health literacy measures to educate patients and their families about the harmful effects of betel quid and TB spread should be implemented at macrolevel agencies with necessary economic and human resources support at downstream agencies. Health care professionals could play a significant role in educating patients. Hence, there is a need to highlight how to effectively utilize physicians, nurses, and public health specialists in this wave to spread awareness focused on preventing the spread of TB in betel nut users. Additionally, regular follow-ups should be conducted with betel users to map their progress and motivate them to quit betel consumption. While there is a lack of empirical evidence on how betel quid consumption can affect TB treatment outcomes, patients should be encouraged to quit betel quid use during the treatment and de-addiction programs; behavior change communication campaigns can aid in this process. There is limited evidence on the role of capacity building among providers, community health workers, and other stakeholders to address this seriously impactful cultural habit. Strategic and time-tested interventions to support the role of capacity building, health literacy, and social partnerships could help us understand what pillars of health systems can be leveraged to address this concern.

### Regulation of Access and Product Availability

Easy access and affordability are other attractive factors explaining why people opt to chew smokeless tobacco such as betel [10, 12]. To curtail this, there is a need to advocate for regulatory and legal measures to control the easy access and affordability of betel. A simulated analysis conducted in Taiwan to identify cross-price elasticity of betel nut based on the tax increases revealed that betel nuts were price inelastic [48]. Conducting simulations of how heavier taxation and legislative regulations could affect betel consumption trends would help in appropriate planning and implementation of regulatory policies. Extents of the chemical components of areca nut in cigarettes that could be used in sports settings (eg, baseball) in countries such as the Dominican Republic and other low- and middle-income countries in the Americas are still unknown; however, on multiple business websites, areca cigarettes are offered at a very low cost, which suggests that they could be used (cigarettes) on a low scale and therefore its right dimensions remain elusive [49, 50].

### Epidemiologic, Longitudinal, and Prospective Research

In addition to the aforementioned measures, it is crucial to advocate for further epidemiologic research to better understand the relationship between betel quid consumption and TB transmission. Epidemiologic studies can help to understand the mechanisms through which betel may contribute to TB risk as well as treatment outcomes, which is crucial for developing strategies in low- and middle-income countries, where there is a dual burden of TB and social factors such as betel quid consumption.

## LIMITATIONS OF THE STUDY

There are extensive variations in cultural practices and geographic differences in betel quid use across regions; hence, it will be difficult to generalize any data and the findings related to this topic. Moreover, there is a shortage of mechanistic studies that investigate the biological mechanisms through which betel quid use might influence TB transmission, hindering a thorough understanding of the underlying processes. Future research addressing these limitations will be essential for achieving a more precise and comprehensive understanding of the connection between betel quid use and TB transmission.

## CONCLUSION

Low- and middle-income countries experience a dual disease burden of TB and betel quid consumption, both of which act as a social determinants and contribute significantly to global health concerns and elevated mortality rates. Global health implications of betel quid consumption necessitate a multipronged approach aimed at raising awareness about its health risks, formulating regulations in accessibility, and conducting research to better understand its implications for TB transmission. Prospective research to determine the potential association of betel quid use and its association with TB transmission as well as its impact on TB treatment outcomes can help build evidence to address the knowledge gap while attending to this missed opportunity in TB control.

## Notes

***Author contributions***. P. M., N. P., N. K.: study design, data synthesis, and manuscript preparation, with revisions and final approval of the manuscript. R. P. R., S. G., K. M. M. I.: study design and manuscript preparation, with revisions and final approval of the manuscript. P. M., T. H., H. D.: manuscript preparation, with revisions and final approval of the manuscript.

***Disclaimer*.** The viewpoints expressed in this article are solely those of the authors and do not reflect the official position or policies of any government agency.

***Patient consent statement*.** The study is a review article and does not include any factors necessitating patient consent.
